# Increased PD-L1 expression in radioresistant HNSCC cell lines after irradiation affects cell proliferation due to inactivation of GSK-3beta

**DOI:** 10.18632/oncotarget.26542

**Published:** 2019-01-15

**Authors:** Daniela Schulz, Irene Stancev, Antonio Sorrentino, Ayse-Nur Menevse, Philipp Beckhove, Gero Brockhoff, Matthias Günther Hautmann, Torsten Erich Reichert, Richard Josef Bauer, Tobias Ettl

**Affiliations:** ^1^ Department of Oral and Maxillofacial Surgery, University Hospital Regensburg, Regensburg, Germany; ^2^ Regensburg Center for Interventional Immunology, University Regensburg and Department of Hematology-Oncology, Internal Medicine III, University Hospital Regensburg, Regensburg, Germany; ^3^ Department of Gynecology and Obstetrics, University Medical Center Regensburg, Regensburg, Germany; ^4^ Department of Radiotherapy, University of Regensburg, Regensburg, Germany; ^5^ Center for Medical Biotechnology, Department of Oral and Maxillofacial Surgery, University Hospital Regensburg, Regensburg, Germany

**Keywords:** PD-L1, immune checkpoint, head and neck cancer, irradiation, GSK-3beta

## Abstract

At present, targeting PD-1/PD-L1 axis for immune checkpoint inhibition has improved treatment of various tumor entities, including head and neck squamous cell carcinoma (HNSCC). However, one part of the patient cohort still shows little improvement or even hyperprogression. We established three radioresistant (RR) and three radiosensitive (RS) HNSCC cell lines. RR cells showed prolonged survival as well as delayed and diminished apoptosis after irradiation with vimentin expression but no E-cadherin expression, whereas RS cell lines died early and exhibited early apoptosis after irradiation and high vimentin expression. Here, we present results demonstrating differential basal PD-L1 gene and protein expression in RR and RS HNSCC cell lines. Moreover, we observed a radiation dose dependent increase of total PD-L1 protein expression in RR cell lines up to 96h after irradiation compared to non-irradiated (non-IRR) cells. We found a significant GSK-3beta phosphorylation, resulting in an inactivation, after irradiation of RR cell lines. Co-immunoprecipitation experiments revealed decreased interaction of GSK-3beta with PD-L1 in non-IRR compared to irradiated (IRR) RR cells leading to PD-L1 stabilization in RR cells. PD-L1 knockdown in RR cells showed a strong decrease in cell survival. In summary, our results suggest an irradiation dependent increase in basal PD-L1 expression in RR HNSCC cell lines via GSK-3beta inactivation.

## INTRODUCTION

With more than 600.000 new diagnosis each year head and neck squamous cell carcinoma (HNSCC) is the 6^th^ most common form of cancer worldwide with a strongly increasing incidence over the last 10 years [1]. Patients suffering from localized HNSCC can be cured by radical surgical resection. In the event of advanced HNSCC, a multidisciplinary approach, including surgical, chemotherapy and radiotherapy, is required. Despite improvement of these therapeutic interventions the survival rate has not increased remarkably over the last years [2]. During recent years immunotherapy by inhibition of checkpoint regulators has become an important part of successful treatment. The PD-1/PD-L1 checkpoint plays a crucial role in the regulation of T-cell activity during inflammatory response to infection controlling autoimmunity. PD-1 has two ligands, PD-L1 and PD-L-2, both of which are members of the B7 family of transmembrane proteins. While the expression of PD-L2 is largely restricted to antigen-presenting cells (APC) APCs, PD-L1 is expressed on many cell types, including T-cells, B-cells, monocytes, APCs and epithelial cells, and is up-regulated in response to proinflammatory cytokines such as IFNγ [3]. Recent studies show that PD-L1 expressing cancer cells have the ability to evade immune response. PD-L1 expression is common in many solid human cancers including colorectal cancer, gastric cancer, esophageal cancer, hepatocellular carcinoma, melanoma, glioblastoma, lung cancer, and oral squamous cell carcinoma [4][5][6][7]. *In vitro* experiments exhibit diminished cancer progression by enhanced T-cell response after inhibition of the interaction between PD-1/PD-L1 [8]. Early clinical trials in patients with recurrent or metastatic head and neck squamous cell carcinoma (HNSCC) using the anti-PD-1 antibodies nivolumab or pembrolizumab demonstrated impressive clinical outcomes for patients, who previously had low prospects on recovery following progression on a platinum-based chemotherapy [9][10]. However, although immune checkpoint inhibition has demonstrated promising results a considerable amount of patients is still showing little improvement or even hyperprogression after PD-1/PD-L1 antibody treatment. Current investigations mainly focus on immunogenic function of a PD-1/PD-L1 interaction. In this context radiotherapy has gained interest as stimulus for CD8^+^ T-cell activation in order to improve sensitivity to cancer immunotherapy [11]. Instead, cellular interactions of PD-L1 in tumor cells are rarely focused [12]. The question whether PD-L1 expression and the associated signaling pathways in tumor cells interfere with molecular events occurring during or after irradiation treatment remains elusive. Recent evidence suggests that PD-L1 can activate intrinsic signals in the absence of PD-1 that enhance tumor cell proliferation and survival [13]. Therefore, in this study we examined PD-L1 expression and cell intrinsic function in radioresistant and radiosensitive HNSCC cell lines before and after irradiation.

## RESULTS

### Radiosensitivity and apoptosis

**Figure 1:**
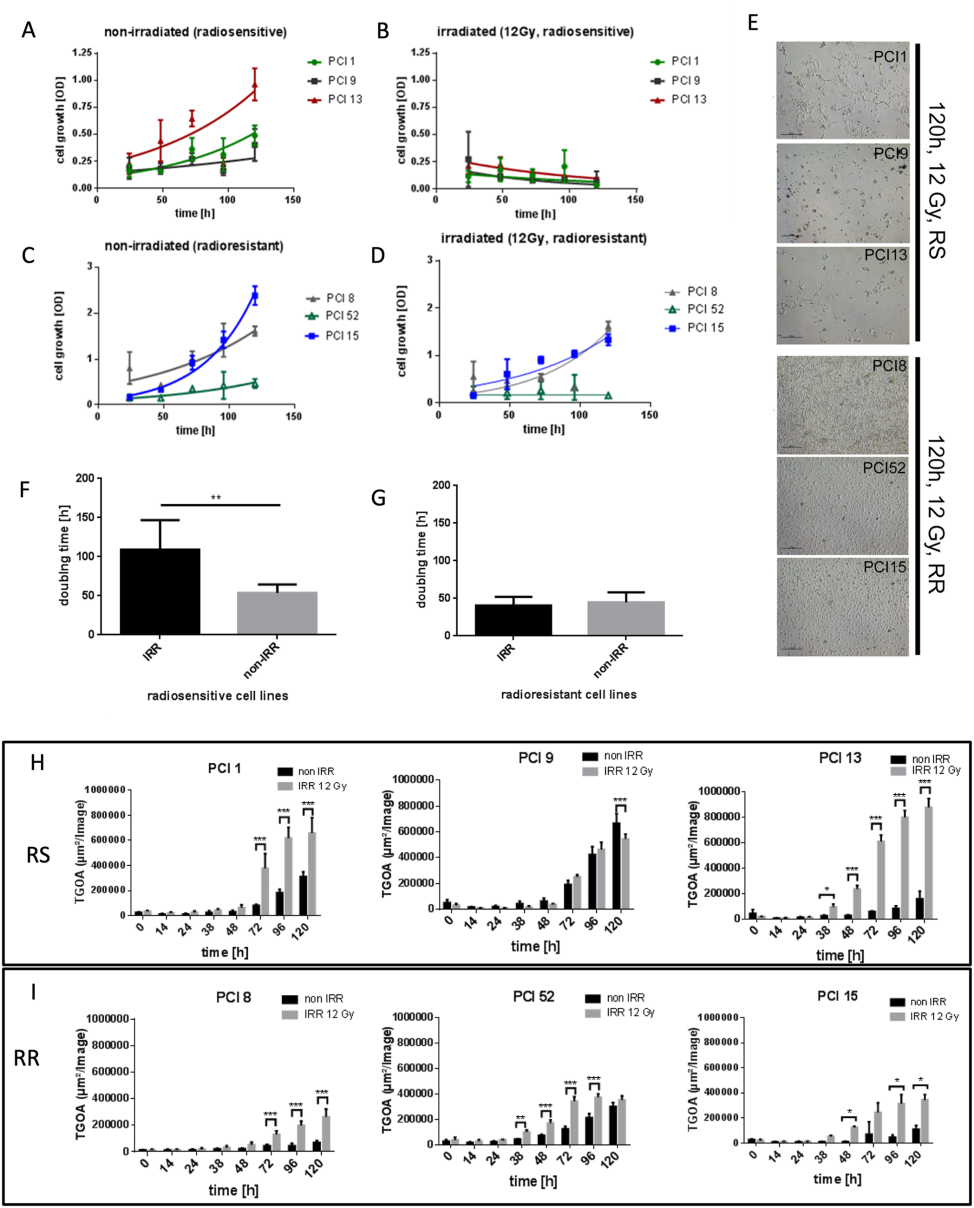
Characterization of radiosensitivity in six HNSCC cell lines via WST-1 viability assay **(A, B)** Viability of RS cell lines 24h – 120h after irradiation with 12Gy. Non-irradiated (non-IRR) cells served as control for unaffected proliferation. Non-IRR controls show constant proliferation during 120h of observation. **(C, D)** Viability of RR cell lines 24h–120h after irradiation. RR cells show proliferation and survival 120h after irradiation. **(E)** Representative images of RS cell lines PCI1, 9, 13 and RR cell lines PCI8, 52, 15, 120h after irradiation. 5 days after irradiation images were taken with 4-fold magnification. RS cell lines were strongly diminished 120h after irradiation, whereas RR cell lines reached confluence of 70% to 100%. **(F, G)** Doubling time of RS and RR cell lines. IRR RS cell lines reacted with extensively prolonged doubling time whereas doubling time of RR cells was unaffected by irradiation. **(H)** Characterization of radiosensitivity via live cell imaging. Each diagram represents the apoptosis rate of a single cell line as total green object area [μm^2^/Image] at depicted time points after irradiation with 12Gy. RS cell lines PCI1, 9, 13 show stronger overall induction of apoptosis compared to RR cell lines PCI8, 15, 52 **(I)**. Black bars represent non-IRR controls (non-IRR). Gray bars represent IRR cells (IRR 12Gy). n=4, Two-Way ANOVA * = p < 0,05, ** = p < 0,01, *** p= < 0,001.

To establish an *in vitro* model for radiosensitivity, HNSCC cell lines were irradiated (IRR) with a dose of 12 Gray (Gy). Cell viability was measured via WST-1 viability assay over a period of 24h – 120h after irradiation. Three cell lines which detached and died within 120h after irradiation were found to be radiosensitive (RS) (PCI1, PCI9, PCI13). Three cell lines which showed proliferation or survival after irradiation were found to be radioresistant (RR) (PCI8, PCI52, PCI15) (Figure 1B, 1D, 1E). Non-irradiated (non-IRR) cell lines served as controls (Figure 1A, 1C). All cell lines exhibited a similar doubling time with a mean of 49.4h in normal non-IRR state (Figure 1F, 1G). After irradiation mean doubling time of RS cell lines PCI1, PCI9 and PCI13 increased to 100.4h whereas doubling time of RR cell lines PCI8, PCI52 and PCI15 remained constant (Figure 1F, 1G). To measure apoptosis in RS and RR cell lines, cells were incubated with the green fluorescent dye YOYO-1 which labels only cells with diminished membrane integrity. The total green object area (TGOA, μm^2^/image) was detected and analyzed via live cell imaging technology over a period of 120h after IRR with one picture per hour. All RS cell lines revealed a strong increase in apoptosis with a minimum of 38h after irradiation and a median green object area of 6, 94x10^5^ μm^2^/image (±1.69x10^5^) 120h after irradiation (Figure 1H). All RR cell lines showed a median green object area of only 2.95x10^5^ μm^2^/image (±0.88x10^5^) with a peak at 96h after IRR (Figure 1I).

### PD-L1 expression in RR and RS cell lines

**Figure 2:**
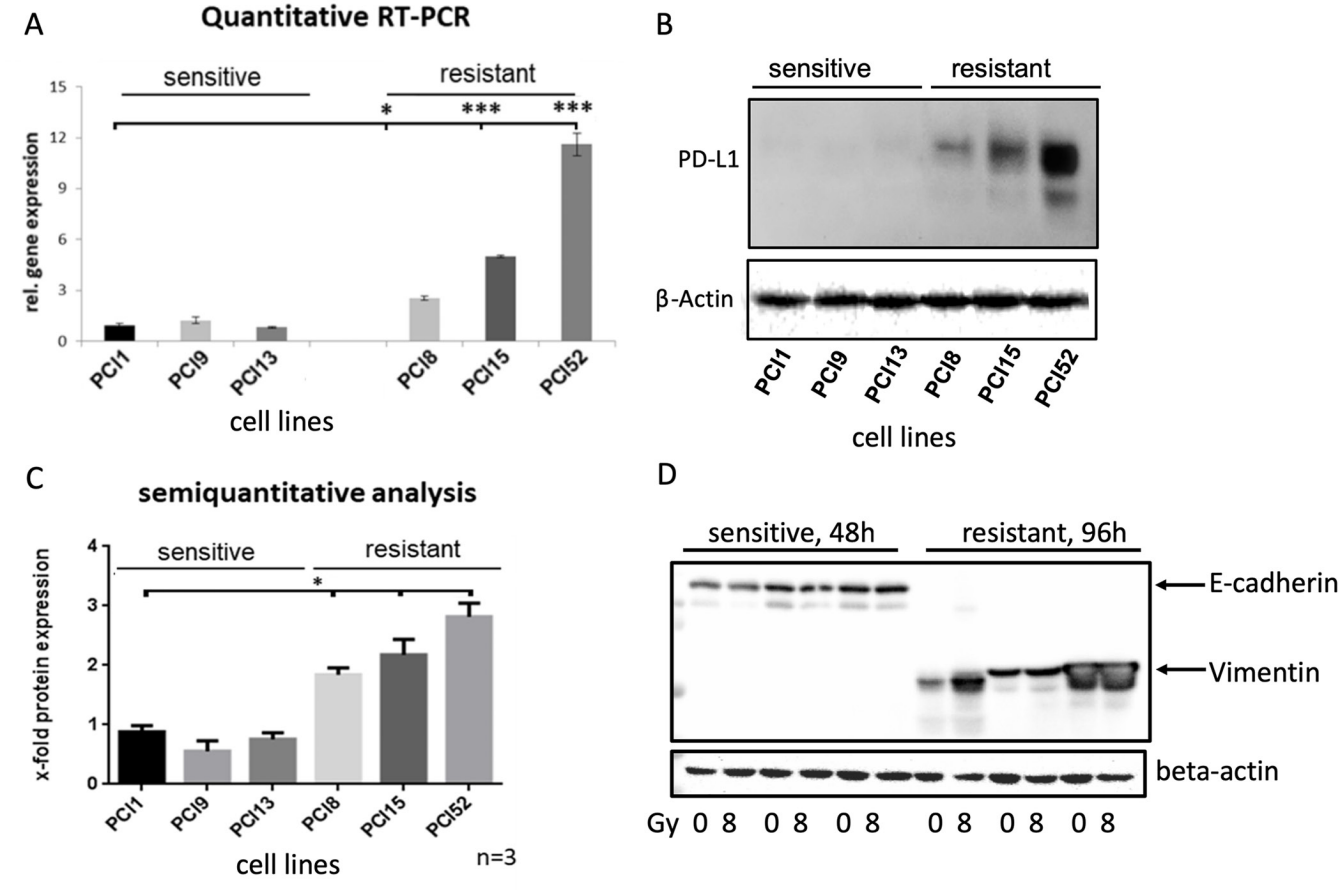
Differential PD-L1 protein expression in HNSCC cell lines with different radiosensitivity **(A)** Quantitative Taqman RT-qPCR showing basal gene expression of PD-L1. No significant difference in PD-L1 gene expression in RS cell lines PCI1, 9, 13, whereas all RR cell lines PCI8, 15, 52 showed significantly higher gene expression of PD-L1 one compared to RS reference PCI1. **(B)** Representative western blot showing basal protein expression of PD-L1. All RR HNSCC cell lines PCI8, 15, 52 show a markedly higher protein expression of PD-L1 than RS cell lines PCI1, 9, 13. **(C)** Semiquantitative analysis of western blot shows a consistent low protein expression of PD-L1 in RS cell lines. No significant difference observable in RS cell lines PCI1, 9, 13, whereas each RR cell lines PCI8, 15, 52 prove a significantly higher protein expression of PD-L1 in comparison to RS reference PCI1. **(D)** Western blot analysis of E-cadherin and Vimentin expression in RS and RR HNSCC cell lines. RS cell lines express E-cadherin but no Vimentin. RR cell lines express Vimentin but no E-cadherin. Lysates from RS cell were taken 48h after irradiation. For quantification the samples derive from the same experiment and blots were processed in parallel. The results are expressed as means ± SD * = p < 0,05, ** = p< 0,01, *** p= < 0.001 when compared to reference control PCI1. n=3, two-tailed Student’s t-Test. (* = p < 0,05).

To analyze PD-L1 gene and protein expression in HNSCC cell lines, we performed Taqman^®^ quantitative RT-PCR and western blot analysis (Figure 2). Surprisingly, non-IRR RR and RS groups depicted differential basal levels of total PD-L1 gene and protein expression. Compared to RS cell lines, all RR cell lines revealed significantly higher PD-L1 gene expression (≥12x, Figure 2A). Moreover, western blots and semiquantitative analysis showed a significantly higher total PD-L1 protein expression (≥3x, Figure 2B, 2C).

Ock et al. reported association between high PD-L1 expression and epithelial-mesenchymal transition (EMT) in head and neck squamous cell carcinoma [14]. We hypothesized that a high PD-L1 expression in our RR cells would result in the expression of EMT marker. In contrast, a low PD-L1 expression would not show EMT markers. To reveal the correlation, it was tested if cell lines express the mesenchymal marker vimentin and the epithelial marker E-cadherin. Western blot analysis was performed with RR and RS cell lines. Interestingly, all RS cell lines expressed full-length E-cadherin but did not show any vimentin expression. In contrast, all RR cell lines revealed a strong vimentin expression but no E-cadherin expression (Figure 2D).

**Figure 3:**
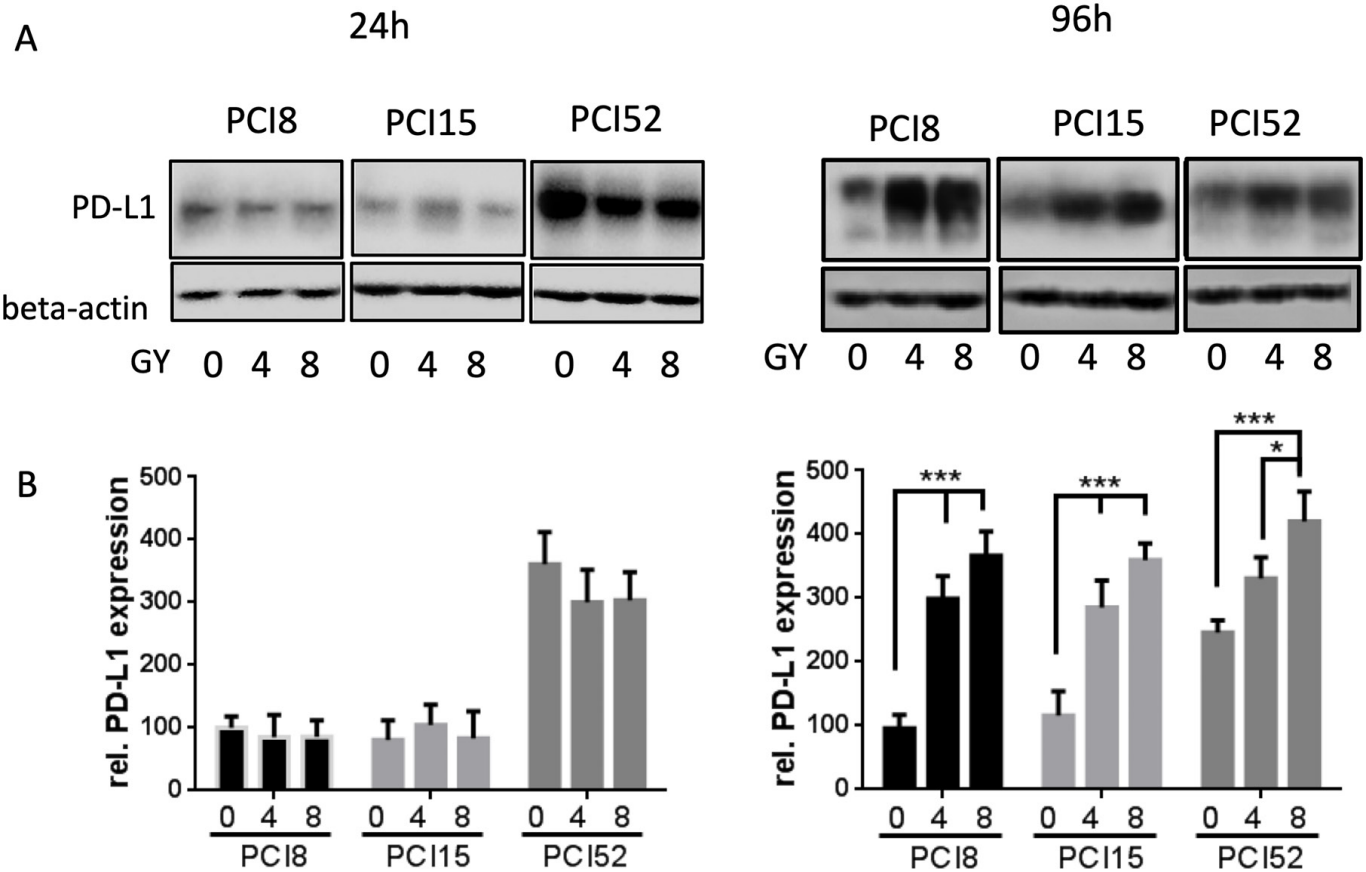
(A) PD-L1 protein expression after irradiation Cells were irradiated with 4Gy and 8Gy. Non-IRR cells served as control (0Gy). 24h and 96h after irradiation cells were lysed in RIPA buffer. Each diagram represents the PD-L1 protein expression of a single cell line. **(B)** Semiquantitative analysis of western blots. 24h after irradiation PD-L1 protein expression hardly changed with increasing radiation. After 96h PD-L1 expression significantly increased dose-dependently. For quantification the samples derive from 3 separate blots where quantifications of the 3 cell lines were processed in parallel. 30µg protein lysate was loaded. n=3, 2-way ANOVA ** = p < 0,01, *** p= < 0,001, samples were normalized with beta-Actin loading control, Non-IRR 0Gy value of PCI8 was used for baseline definition.

To examine if IRR had any influence on PD-L1 protein expression RS and RR cell lines were irradiated with 4Gy and 8Gy. 24h and 96h after irradiation PD-L1 protein expression was examined via immunoblotting (Figure 3A). 24h after irradiation all RR cell lines showed almost equal amounts of PD-L1 protein expression levels, irrespective of irradiation (0Gy, 4Gy or 8Gy). However, 96h after irradiation there was a significant 3 – 4-fold dose dependent increase of PD-L1 protein expression after irradiation (Figure 3B). RS cells did not show any change or increase in PD-L1 expression level 24h after irradiation (data not shown). Data of RS cells 96h after irradiation could not be retrieved because the majority of RS cell lines are no longer viable after this time.

**Figure 4:**
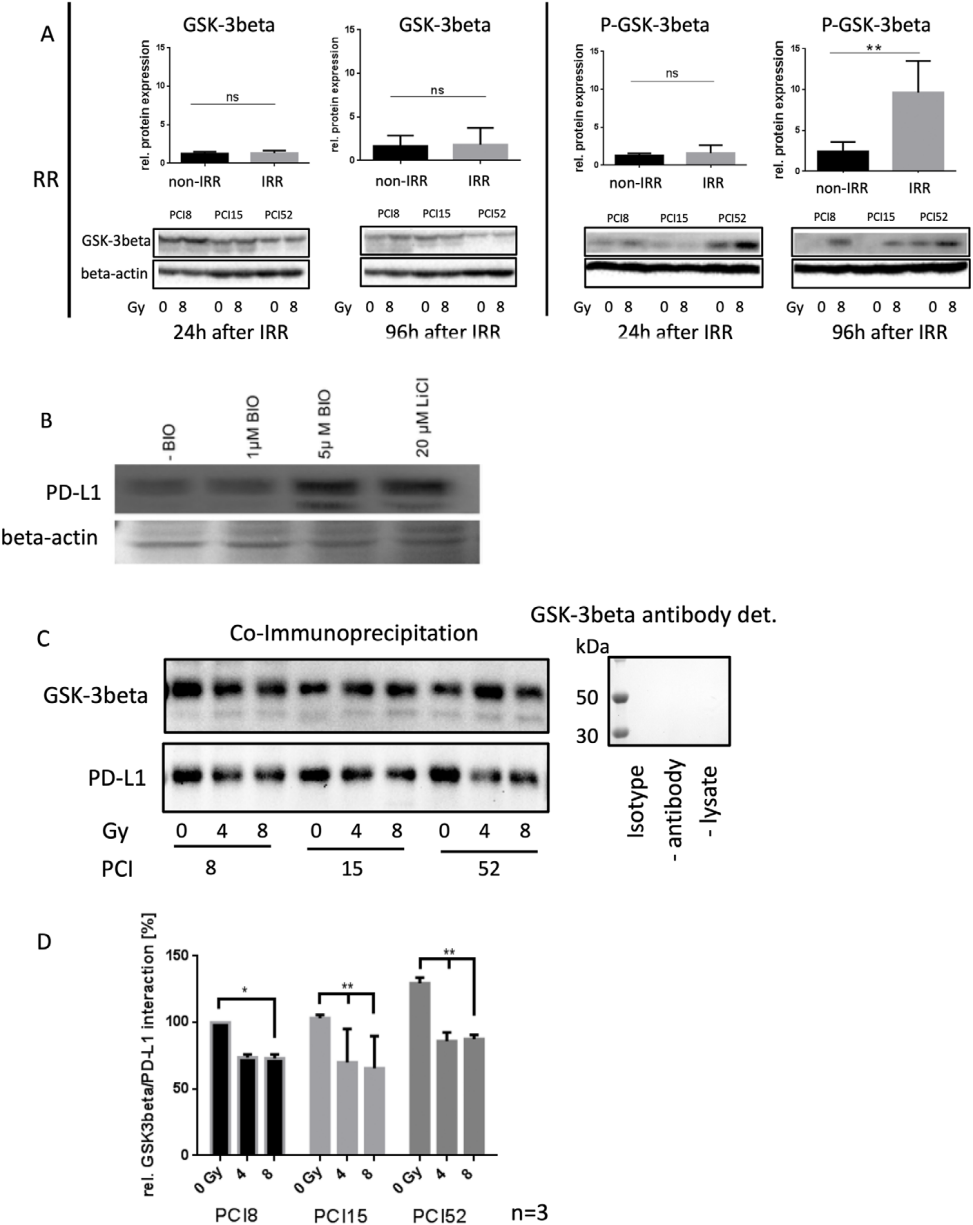
Regulation of PD-L1 protein expression **(A)** Western blot and semiquanititative analysis of GSK-3beta and its inactivated state represented as phosphorylated-GSK-3beta (P-GSK-3beta) at Ser09 24h and 96h after irradiation with 8Gy. Non-IRR cells (0Gy) were used as controls. RR HNSCC cell lines did not show any significant change in GSK-3beta expression (lower left panel). However, inactivation of GSK-3beta occured 96h after irradiation. Phosphorylation at Ser09 was on average 10x higher compared to the level of non-IRR cells. Beta-Actin was used as loading control. n=3, Student’s t-Test ** = p < 0,01, ns=not significant **(B)** PD-L1 expression is dependent on GSK-3beta activation. Western blot analysis of PD-L1 expression after inhibition of GSK-3beta with 20μM LiCl and specific inhibition with 1μM and 5μM BIO. Exemplified GSK-3b dependent PD-L1 expression with HNSCC cell line PCI52. n=3. Ponceau staining was used as loading control. **(C)** Co-immunoprecipitation for identification of an interaction between PD-L1 and GSK-3beta without (0Gy) and after 96h of irradiation with 4 and 8Gy. GSK-3beta, including its attached binding partners, was precipitated with a specific antibody. Presence of PD-L1, necessarily interacting with GSK-3beta, was proven via western blot analysis. All RR cell lines PCI8, 15, 52 showed a decrease of PD-L1 interaction after irradiation. GSK-3beta detection served as loading control. Either a polyclonal rabbit IgG Isotype antibody, immunoprecipitation without antibody or without lysate was used as negative control. Undetectable GSK-3beta indicates no unspecific binding. **(D)** Semiquantitative analysis of western blot indicating relative interaction of GSK-3beta with PD-L1 in percent. After irradiation with 8Gy all RR cell lines PCI8, 15, 52 showed less interaction between GSK-3beta and PD-L1. PCI15 and PCI 52 also showed a significantly reduced interaction of GSK-3beta with PD-L1 after irradiation with 4Gy. Samples were normalized with GSK-3beta. Non-IRR 0Gy value of PCI8 was used for baseline definition. n=3, 2-way ANOVA * = p < 0,05, ** p= < 0,01.

It is known from the literature that PD-L1 is destabilized via binding to active GSK-3beta phosphorylation and subsequent proteasomal degradation of PD-L1 [12]. Therefore, the activation state of GSK-3beta was analyzed in RR and RS cell lines before and after irradiation. We found significantly increased levels of Ser09 phosphorylated GSK-3beta in all RR cell lines (Figure 4A). GSK-3beta was inactivated in RR cell lines after irradiation compared to RS cell lines which did not show altered Ser09 phosphorylation before and after irradiation. This inactivation of GSK-3beta in RR cell lines after irradiation is supposed to stabilize PD-L1 expression.

To find out if PD-L1 regulation is dependent on GSK-3beta, GSK-3beta activation was blocked with an unspecific (LiCl) and a specific (BIO) GSK-3beta inhibitor (Figure 4B). When the GSK-3beta activation was blocked, PD-L1 immunodetection was increased.

To find out, if GSK-3beta directly interacts with PD-L1 in our RR cell lines a co-immunoprecipitation experiment was performed 96h after irradiating the cells with 0Gy, 4Gy and 8Gy. The immunoprecipitation with an antibody targeting GSK-3beta, was subjected to western blot analysis with subsequent PD-L1 immunodetection (Figure 4C). The experiments revealed a strong interaction of GSK-3beta in cells without irrradiation. However, increasing irradiation repeatedly diminished the interaction of GSK-3beta with PD-L1 (Figure 4D).

**Figure 5:**
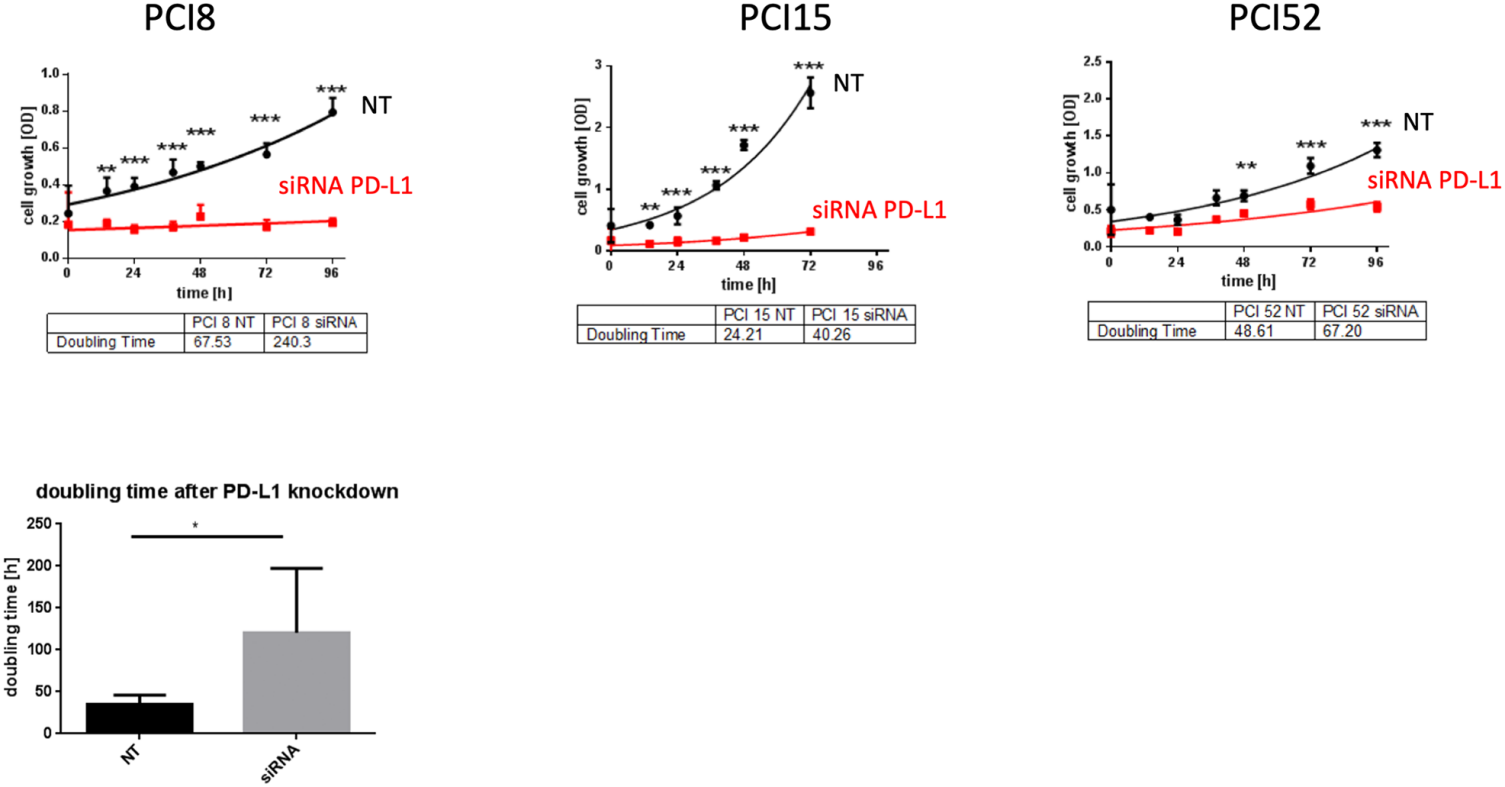
PD-L1 function in RR cell lines **(A)** WST-1 viability assay after siRNA knockdown of PD-L1 in RR HNSCC cell lines. All cell lines with PD-L1 knockdown showed a substantial decrease of proliferation compared to cells transfected with scrambled siRNA (NT) as control during the course of 96h. **(B)** The average doubling time of PD-L1 knockdown cells was 115,9h compared to NT control cells with 46,8h. NT= non-targeting, scrambled non-specific siRNA. Two-way ANOVA * = p < 0,05, ** = p < 0,01, *** = p < 0,001.

To examine PD-L1 function in RR HNSCC cell lines we performed a siRNA knockdown experiment. Cell proliferation was measured via WST viability assay. Figure 5 presents a significant decrease of cell viability after PD-L1 siRNA knockdown in all RR HNSCC cell lines during the course of 96h compared to cells treated with non-targeting (NT) scrambled siRNA as control. The average doubling time of PD-L1 knockdown cells was 115, 9h compared to NT cells with 46, 8h.

## DISCUSSION

There was a high basal PD-L1 gene and total protein expression in all RR cell lines which was 2.7-fold higher compared to all RS cell lines. Müller et al. observed high PD-L1 expression in a subset of HNSCC and detected a strong correlation between PD-L1 expression and overall survival. Moreover, PD-L1 is supposed to be a prognostic factor of adverse patient outcome in HNSCC being associated with the presence of distant metastases [15]. In tumor tissues from 50 patients with HNSCC 64% were PD-L1 positive. In these tissues PD-L1 expression was significantly associated with EMT as assessed by low E-cadherin and high vimentin expression [16]. This conforms with our data showing no E-cadherin protein expression but high vimentin expression in all RR cell lines compared to RS cell lines which all expressed E-cadherin but no vimentin. Li et al. showed interaction of GSK-3beta with PD–L1 with subsequent phosphorylation dependent proteasome degradation of PD–L1 by beta-TrCP [12].

Radiation modulates the expression of immune checkpoint ligands, including PD-L1, on the surface of tumor cells and on immune cells in the tumor microenvironment [16][17]. Dovedi and colleagues studied the effect of radiation combined with anti-PD-1 and anti-PD-L1 antibodies. Combined treatment generated efficacious CD8^+^ T-cell responses that improved local tumor control in murine colon and breast cancer models. This group noted that natural killer cells might contribute to local tumor control combination therapy, but long-term survival is dependent on CD8^+^ T cells [16]. PD-L1 signaling seems to be associated with radioresistance also in head and neck cancer. Tumors with high expression of PD-L1 had failure rates following radiotherapy of 50%-70% compared to 20%-25% with low PD-L1 expression [18]. Moreover, preclinical and clinical data support the potential immunologic synergy of irradiation and immune checkpoint blockade. Deng et al., described a reduction of local accumulation of tumor infiltrating myeloid-derived suppressor cells in mice. Significantly improved local tumor control was reported when irradiation was combined with anti-PD-L1 in mouse models of breast and colorectal cancer [19]. The synergistic effect of radiosensitizing chemotherapy was also observed in non-small cell lung cancer (NSCLC) [20]. Additionally, recent data by Quin et al. showed that radiotherapy combined with PD-L1 inhibition leads to complete local and abscopal response in refractory Hodgkin`s Lymphoma [21]. The majority of studies focus on combined systemic immunological effects of radiotherapy and the PD1/PD-L1 axis via CD8 or MHC. However, our data suggest a strong effect of irradiation on PD-L1 expression and a subsequent functional impact on cellular level. Recent evidence suggests that PD-L1 can activate intrinsic signals in the absence of PD-1 that enhance cell proliferation and survival through the inhibition of autophagy and target of rapamycin (mTOR) activation [13][22][23]. Moreover, Zhao et al. demonstrated a correlation of phosphatase and tensin homologue (PTEN) with overexpression of PD-L1 in pancreatic cancer tissue. Loss of PTEN aberrantly activates phosphatidylinositol 3-kinase/Akt/mammalian target of rapamycin (mTOR) and thereby promotes survival and proliferation [24]. In our cell lines 24h after irradiation with 8Gy there were equal expression levels of PD-L1, both in non-IRR and IRR RR HNSCC cell lines. In PCI52 PD-L1 expression even seems to slightly decrease with 4Gy and 8Gy of irradiation. Comparing the non-IRR cells 24h and 96h PCI8 and PCI15 have about equal amount of PD-L1 expression. In non-IRR PCI52 PD-L1 rather decreases. Interestingly, 96h after irradiation we observed a substantial increase of PD-L1 in IRR cells compared to non-IRR cells. This could be due to *de novo* synthesis or to stabilization of PD-L1 in IRR RR HNSCC cells. A Taqman qPCR experiment partly showed an increase of *de novo* gene expression after irradiation. Besides increased *de novo* expression, our hypothesis that PD-L1 might be stabilized after irradiation was supported by immunoblot data showing increased phosphorylation, i.e. inactivation, of GSK-3beta 96h after irradiation of RR cell lines. In addition, our data suggest a decrease of GSK-3beta and PD-L1 interaction 96h after irradiation with 4Gy and 8Gy compared to non-IRR HNSCC cells. To address the function of PD-L1 in IRR cells knockdown experiments were performed in RR cells with siRNA against PD-L1. The results revealed a strong decrease in cell proliferation. This suggests involvement of PD-L1 expression in cellular proliferation activity. Therefore, PD-L1 involvement in cell proliferation might support the survival of IRR RR HNSCC cells. Further experiments have to reveal the mechanisms and the connection of PD-L1 expression and HNSCC cell proliferation. Clark et al. revealed tumor-intrinsic PD-L1 signals regulating cell growth, pathogenesis and autophagy in two distinct models, ovarian cancer and melanoma [23]. These tumors arise in two distinct anatomic compartments and the effect on proliferation was shown to be immune-independent [23]. Xue et al. reported a positive correlation of PD-L1 expression and Ki-67 expression levels in glioma [25]. Moreover, overexpression of miR-140 was shown to suppress PD-L1 expression with decreased cyclin E expression in NSCLC. Inhibition of PD-L1 also decreased the expression of cyclin E [26]. In summary, our data show an increased basal expression level of PD-L1 in RR HNSCC cells. Moreover, additional to *de novo* expression, our data suggest a longer turnover time of PD-L1 in IRR RR cells due to GSK-3beta inactivation and subsequent PD-L1 stabilization. This stabilization in turn might exert an intrinsic survival advantage facilitating HNSCC cell proliferation. For radioresistant recurrent tumors this would imply, that a combination therapy targeting different signaling axes could potentially have synergistic effects in tumor therapy. Long term goal for the project is also to examine the localization of PD-L1 in tumor cells with different molecular baseline equipment.

## MATERIALS AND METHODS

### Cell lines and culture conditions

The human HNSCC cell lines PCI1, PCI8, PCI9, PCI13, PCI15, PCI52 were kindly provided by Prof. Dr. Theresa. L. Whiteside (University of Pittsburgh Cancer Institute (PCI), Pittsburgh, PA). The cell lines were established from primary tumors of different origin in the laboratory at the University of Pittsburgh: PCI1—larynx, PCI8—Pyriform, PCI9—base of tongue, PCI13—retromolar triangle, PCI15—Pyriform fossa, PCI52—Plica aryepiglottica [27] [28]. HNSCC cell lines were maintained in DMEM (PanBiotech, Aidenbach, Germany) supplemented with 10% fetal calf serum (FCS, Gibco, Carlsbad, CA, USA), 1% L-glutamine (Sigma-Aldrich, Munich, Germany) and 1% penicillin/ streptomycin (Sigma-Aldrich, Munich, Germany) at 37°C in a 5% CO_2_humidified atmosphere. The medium was changed every two to three days and the cells were passaged prior reaching confluence. Cells were detached by incubation with 0,05% trypsin-EDTA solution (Sigma-Aldrich, Munich, Germany) for 5 to 10 minutes (min) at 37°C.

### Cell irradiation

Culture plates were placed on the acceleration treatment couch. In order to compensate for the build-up effect, 2-cm thick plates of perspex were positioned above and below the tissue culture vials. As previously described by Pohl et al. the external irradiation was delivered via an anterior portal by a 6-MV linear accelerator emitting a photon beam (3 Gy/min; Primus, Siemens, Clin Oral Invest Nuernberg, Germany) at room temperature [29]. Cells were irradiated not earlier than 24h after seeding. Immediately after radiation the culture medium was replaced by fresh medium. Non-IRR cells served as control.

### Viability assay

Cell proliferation was measured *in vitro* using the WST-1 proliferation assay kit according to manufacturer`s instructions (Sigma Aldrich, Munich, Germany). 2000 cells were seeded into a 96-well plate (Greiner Bio-One, Kremsmünster, Austria) and cultured for 24h. Immediately after irradiation with 12Gy the culture medium was replaced by fresh medium. Cell proliferation was measured 24, 36, 48, 72, 96 and 120h after irradiation. 10μl of WST-1 reagent was added to 100μl of the culture medium. Cells were incubated with the WST-1 reagent for 3h. The amount of soluble formazan dye formed directly from tetrazolium salt WST-1 correlates to the number of metabolically active cells in the culture. Culture medium with WST-1 reagent in the absence of cells was used as a background control. Absorbance (optical density (OD) was measured at 450 nm using a spectrophotometer. To maintain optimal and constant growth conditions at each time point, every 24h, half of the culture medium was replaced by fresh medium.

**Table:** 

Gene	Name	Primer-Sequence (5’-3’)	UPL-Probe
18S	P208 for	GCAATTATTCCCCATGAACG	#48
	P208 rev	GGGACTTAATCAACGCAAGC	#48
PD-L1 / CD274	P213 for	CTACTGGCATTTGCTGAACG	#48
	P213 rev	TGCAGCCAGGTCTAATTGTTT	#48

### Apopototic measurement by means of live cell imaging

Live cell imaging assays were performed with live cell IncuCyte Zoom automated imaging system (Essen BioScience, Ann Arbor, MI, USA). 2000 cells were seeded onto black 96-well plates with clear bottom and cultured for 24h. Subsequently after irradiation the culture medium was replaced by fresh medium, containing YOYO-1 (Life Technologies, Carlsbad, CA, USA), a fluorescent dye for labelling apoptotic cells. This membrane-integrity based cytotoxicity assay measures the uptake of YOYO-1, which is normally excluded from intact cells. The dye is a cell impermeant cyanine dimer nucleic acid stain that binds to dsDNA which allows for the kinetic evaluation of cytotoxicity. Cells were also monitored morphologically and quantified using the IncuCyte ZOOM basic analyzer. The plates were incubated for 120h and imaged (10x magnification) at regular intervals of 1h. Phase-contrast or fluorescence images were gathered and processed to determine confluence (as percentage of the covered area) and cell death (as total green object area [μm^2^/Image]) over time. All experiments were performed without lifting or washing so are not perturbing to the cell model. A non-IRR plate was used as a control.

### Gene expression

Total cellular RNA from cells was isolated using the RNeasy mini kit (Qiagen, Hilden, Germany) according to the manufacturer’s instructions. Reverse transcription of 1μg RNA to complementary DNA (cDNA) was performed using transcriptor First Strand cDNA synthesis kit (Roche, Mannheim, Germany) according to the manufacturer’s protocol. cDNA was amplified using the Brilliant III ultra fast quantitative polymerase chain reaction master mix (Stratagene Agilent Technologies, Santa Clara, CA.) in combination with TaqMan UPL probes (Roche, Mannheim, Germany) Nr. 48. Real-time PCR primers were obtained from TIB Molbiol (Berlin, Germany). 18S messenger RNA (mRNA) was used for normalization. Following primers were used for quantification.

### Protein expression

Cells were washed with phosphate-buffered saline (PBS, Sigma-Aldrich, Munich, Germany) and harvested by scraping in radioimmunoprecipitation assay buffer (RIPA, Sigma-Aldrich, Munich, Germany) lysis buffer containing protease inhibitor (Roche, Mannheim, Germany). The protein concentration was determined by bicinchoninic acid assay (Merck, Darmstadt, Germany). 30μg total protein was denatured at 70°C for 10 min in Laemmli sample buffer (Bio-Rad, Hercules, CA, USA) containing 1% β-Mercaptoethanol (Merck, Darmstadt, Germany). Samples were were separated by SDS-PAGE using a 10% resolving gel and transferred onto PVDF membrane (Roche, Mannheim, Germany). Blots were blocked in 3% BSA (Biomol, Hamburg, Germany) or 5% skimmed milk (Roth, Karlsruhe, Germany) in PBS containing 0,1% Tween 20 (Sigma-Aldrich, Munich, Germany) for 1h at room temperature followed by incubation with primary antibody at 4°C overnight. Antibodies used for western blot analysis were Phospho-GSK-3beta (Ser09) (D85E12) rabbit mAb #5558 Cell Signaling, GSK-3beta (D5C5Z) XP® rabbit mAb #12456 Cell Signaling, PD L1 (E1L3N) rabbit mAb #13684 Cell Signaling, E-cadherin mouse mAb 610182 BD Bioscience and vimentin rabbit mAB #5741 Cell Signaling.

After washing, membranes were incubated with secondary antibodies conjugated with horseradish peroxidase (HRP), goat anti rabbit stabilized peroxidase conjugated (32460 Thermo Fisher Scientific), goat anti mouse stabilized peroxidase conjugated (32430 Thermo Fisher Scientific). For protein detection Roti Lumin (Roth, Karlsruhe, Germany) or Supersignal WestFemto (Thermo Scientific, Bonn, Germany) was used. Colorimetric and chemiluminescent pictures were processed with the high-resolution, high-sensitivity ChemiDoc XRS+ Imaging System (Bio-Rad, Hercules, CA, USA). Equal loading was verified with mouse antibody against β-actin rabbit polyAb ab8227 Abcam. All experiments were repeated at least three times. Samples were analyzed with the Image Lab software 5.2.1 (Bio-Rad, Hercules, CA, USA). Prior to beta-actin antibody incubation on the same membrane the respective primary antibody was effectively removed from western blots using ReBlot Plus Strong Antibody Stripping Solution (Merck, Darmstadt, Germany). For semiquantitative evaluation the contrast of whole images was slightly enhanced.

### Co-immunoprecipitation

Co-immunoprecipitation was performed using MACS Technology (Miltenyi Biotec, Bergisch Gladbach, Germany) according to the manufacturer’s instructions. 50μg μMACS Protein A MicroBeads (Miltenyi Biotec, Bergisch Gladbach, Germany) were incubated with 1μg binding antibody GSK-3beta (D5C5Z) XP® rabbit mAb #12456 Cell Signaling) and 200μg RIPA-Lysate for 30 min. Incubation with either a polyclonal rabbit IgG Isotype antibody, without antibody or without lysate used as negative controls for unspecific binding. μColumns (Miltenyi Biotec, Bergisch Gladbach, Germany) were placed in the thermoMACS Separator (Miltenyi Biotec, Bergisch Gladbach, Germany). After equilibration of columns the mix was transferred to the columns. Magnetic beads attached to columns and non-bound proteins were discarded. Proteins bound to magnetic beads have been eluted with hot (95°C) Laemmli sample buffer (Biorad, Hercules, CA, USA) containing 1% β-Mercaptoethanol (Merck, Darmstadt, Germany). For denaturation samples were put at 95°C for 5 min. Afterwards SDS-page and western blot analysis were performed as previously described. PD-L1 was detected using PD-L1 (E1L3N, rabbit mAb #13684 Cell Signaling) primary antibody. After membrane stripping equal loading was verified with GSK-3beta (rabbit mAb #12456 Cell Signaling). After washing, membranes primary antibodies were incubated with rabbit monoclonal TrueBlot secondary antibody (Rockland Immunochemicals Inc., Limerick, PA, USA) conjugated with horseradish peroxidase (HRP). The secondary antibody designed for immunoprecipitation with magnetic beads does not detect any of the denatured heavy and light chain of the immunoprecipitation antibody. For protein detection Roti Lumin (Roth, Karlsruhe, Germany) was used. All experiments were repeated at least three times. Samples were analyzed with the Image Lab software 5.2.1.

### Inhibition of GSK-3beta

PCI52 cells were grown to 70% confluency and incubated with the specific GSK-3beta inhibitor BIO (Sigma-Aldrich, Germany) for 24h. Additionally the cells were incubated with 20 mM lithium chloride (Merck, Darmstadt, Germany) for 24h. Then the cells were lysed with RIPA and prepared for SDS gel electrophoresis.

### Transient PD-L1 knockdown via siRNA transfection

To deplete PD-L1 expression, cells were transiently transfected with small interfering RNA (siRNA) specifically against human PD-L1 using Dharmafect-1 transfection reagent (Thermo Scientific, Bonn, Germany), according to the manufacturer’s instructions. For a reverse transfection 2,5^*^10^5^ freshly passaged cells are added to pre-plated transfection complexes in six-well plates (Corning Costar, Germany). The transfection with 25 nM siRNA was performed in DMEM (with 1% L-glutamine and 10% FCS, without antibiotics) for 72h.

The siGENOME Human CD274 (29126) siRNA- SMARTpool (M-015836-01-0005) consisted of four siRNA target sequences: UGAAAGGACUCACUUGGUA (D-015836-01), CAUAGUAGCUACAGACAGA (D-015836-02), AGACCUGGCUGCACUAAUU (D-015836-03), GGACCUAUAUGUGGUAGAG (D-015836-04).

ON-TARGETplus Non-Targeting Pool (D-001810-10-20) consisted of four siRNA target sequences: UGGUUUACAUGUCGACUAA, UGGUUUACAUGUUGUGUGA, UGGUUUACAUGUUUUCUGA, UGGUUUACAUGUUUUCCUA, UGGUUUACAUGUUUUCCUA.

Three days after siRNA transfection, cells were used for experiments. The expression of PD-L1 in HNSCC cell lines was determined at the beginning of the experiment and 96h later by RT-qPCR and western blot analysis. At the beginning of the experiment siRNA transfected cells showed a PD-L1 reduction of ±80%. Transient siRNA knockdown was efficient for at least the duration of the observation.

### Statistical analysis

Statistical analysis was performed using GraphPad Prism 6 Software (GraphPad Inc., San Diego, CA, USA). Results are presented as mean± standard deviation (SD) Each assay was performed in replicates and repeated in three to six independent experiments. Comparisons between two groups were done using the Student's unpaired t-test. The influence of different categorical independent variables on one continuous dependent variable were analyzed using two-way ANOVA. A p-value ≤ 0.05 was considered to be statistically significant.
